# Evolutionary Understanding of Metacaspase Genes in Cultivated and Wild *Oryza* Species and Its Role in Disease Resistance Mechanism in Rice

**DOI:** 10.3390/genes11121412

**Published:** 2020-11-26

**Authors:** Ruchi Bansal, Nitika Rana, Akshay Singh, Pallavi Dhiman, Rushil Mandlik, Humira Sonah, Rupesh Deshmukh, Tilak Raj Sharma

**Affiliations:** 1 National Agri-Food Biotechnology Institute (NABI), Mohali, Punjab 140306, India; ruchibansal18@gmail.com (R.B.); siesta.nitika@gmail.com (N.R.); akshaybioinfo@gmail.com (A.S.); pallavidhiman007@gmail.com (P.D.); rushilmandlik91@gmail.com (R.M.); biohuma@gmail.com (H.S.); 2 Department of Biotechnology, Panjab University, Chandigarh 160014, India; 3 Department of Crop Science, Indian Council of Agriculture Research (ICAR), Krishi Bhavan, New Delhi 110001, India

**Keywords:** abiotic stress, evolution, gene expression, haplotype diversity, *Oryza* species, rice blast

## Abstract

Metacaspases (MCs), a class of cysteine-dependent proteases found in plants, fungi, and protozoa, are predominately involved in programmed cell death processes. In this study, we identified metacaspase genes in cultivated and wild rice species. Characterization of metacaspase genes identified both in cultivated subspecies of *Oryza sativa*, *japonica*, and *indica* and in nine wild rice species was performed. Extensive computational analysis was conducted to understand gene structures, phylogenetic relationships, *cis*-regulatory elements, expression patterns, and haplotypic variations. Further, the haplotyping study of metacaspase genes was conducted using the whole-genome resequencing data publicly available for 4726 diverse genotype and in-house resequencing data generated for north-east Indian rice lines. Sequence variations observed among wild and cultivated rice species for metacaspase genes were used to understand the duplication and neofunctionalization events. The expression profiles of metacaspase genes were analyzed using RNA-seq transcriptome profiling in rice during different developmental stages and stress conditions. Real-time quantitative PCR analysis of candidate metacaspase genes in rice cultivars Pusa Basmati-1 in response to *Magnaporthe oryzae* infection indicated a significant role in the disease resistance mechanism. The information provided here will help to understand the evolution of metacaspases and their role under stress conditions in rice.

## 1. Introduction

Plants are able to engage in programmed cell death, which can be defined as a schematic mode of action that mediates cell death both under stress and at developmental phases of life [1,2]. The phenomenon of programmed cell death is thoroughly characterized in higher animals where caspases are found to have the key role [3,4]. In contrast, very little is known about the programmed cell death in plants and fungi, which lack caspases [5]. Instead of caspases, metacaspases and paracaspases, which are structurally related to caspases, are present in plants and fungi [6]. The metacaspases and paracaspases belong to the C14 family of proteases. Interestingly, metacaspases can be found in all eukaryotic lineages except for a strict absence in animals [7]. Numerous studies have been performed to characterize the metacaspases to understand structural and functional features defining their precise role [8,9,10,11].

Structurally, metacaspases contain two domains, p20 and p10. The metacaspases’ catalytic activity is due to the presence of conserved cysteine and histidine residues in the p20 domain [12]. Along with p20 and p10 domains, additional domains are present in the structure of metacaspases. On the basis of these additional domains, metacaspases are classified into Type I and Type II groups [8,13]. Similar to caspases in animals, a proline-rich prodomain is present in the N-terminal region of Type I metacaspases, which is known to be responsible for inflammation and initiation of programmed cell death. Another prodomain known as LSD-1 (Lysine-specific histone demethylase-1), LOL zinc fingers are present in the structure of some type I metacaspases. Rice *metacaspase 1 OsMC1* and *OsMC3* interact strongly with this domain in regulating programmed cell death [14]. Both of these prodomains are absent in Type II metacaspases. Additionally, metacaspase type II has an elongated linker region present between the p10 and p20 domains, which is absent in Type I metacaspases.

Genome-wide studies have been performed to understand the evolution of metacaspase gene families in plant species having whole-genome sequence resources. In the model plant *Arabidopsis*, nine metacaspase genes were identified, among which three (*AtMC1* to *AtMC3*) belong to Type I and six (*AtMC4* to *AtMC9*) belong to Type II [15]. The effect of type II metacaspase *AtMCP2d* (different nomenclature) in response to the fumonisin mycotoxin B1 in the *Arabidopsis* plant system has been studied [16]. The over-expression of *AtMCP2d* showed accelerated cell death in response to pathogen attack, whereas the *mcp2d-1* mutant showed reduced sensitivity and low induction of oxidative stress [16]. Similar to *AtMC9*, overexpression of metacaspase gene *Camc9* was reported to enhance pathogen-induced programmed cell death in *Capsicum annuum* [17]. The role of metacaspases in boron-induced programmed cell death was studied in barley. The expression of *MC4* in barley was reported to be significantly increased, whereas the expression of *MC5* was antagonistic to *MC4* and was downregulated in barley [18]. Similarly, the role of Type II metacaspase gene *TaMCA4* in wheat was studied for hypersensitive plant death in response to *Puccinia striiformis*. The expression level of *TaMCA4* in wheat leaves was highly upregulated in response to *P. striiformis* infection [19]. In response to the fungal pathogen, *Botrytis cinerea,* the expression of Type II metacaspase *LeMCA1* in tomato was reported to be rapidly increased [20]. Numerous studies conducted over a period of time established the role of metacaspase genes in plant defense when exposed to biotic or abiotic stresses.

The current study is conducted to analyze the role of metacaspases in wild and cultivated *Oryza* species. Due to their adaptation in different conditions over time, the wild rice species possess an immense potential to solve many grueling problems concerning yield, disease resistance, drought, and salt tolerance and, hence, are considered an important aspect of studying the molecular evolution of resistance- and yield-related genes [21,22,23]. Introgression of the Xa21 resistance to bacterial blight from *Oryza longistaminata* is a brilliant illustration of the advantage of exploiting wild genomes [24]. In around 15 million years, 27 *Oryza* species have evolved into 11 types of genomes, and among those, six of them are diploid with a total chromosomal count of 12 (AA, BB, CC, EE, FF, GG), whereas the other five are allotetraploid (2n = 4x = 48) (BBCC, CCDD, HHJJ, HHKK, KKLL) [25,26]. The gene pool of the cultivated rice species *Oryza sativa japonica* is the AA type, and hence to study the metacaspase genes in other *Oryza* clades, ten rice species were characterized. In this study, we analyzed the variation in structural, biochemical, and functional divergence of metacaspases in cultivated and wild rice species.

## 2. Materials and Methods

### 2.1. Identification and Nomenclature of Rice Metacaspase Genes Sequences

The protein sequences of *O. sativa* subspecies *japonica* and *indica,* and nine other wild *Oryza* species were retrieved from EnsemblPlant [27]. The wild species having AA genome similar to *O. sativa* include, *O. nivara*, *O. barthii*, *O. glumaepatula*, *O. glaberrima*, *O. longistaminata*, and *O. rufipogon*, whereas two species, *O. punctata* and *O. meridionalis*, have BB genome, and only one species *O. brachyantha* has FF genome. The *O. nivara* as a separate species is not yet established, but considering the whole genome resequencing of reference genome, here we considered it as a species. The retrieved protein data were used to create a local database in BioEdit [28]. Subsequently, BLASTp search was performed using nine metacaspase genes (*AthMC1–AthMC9*) reported in *Arabidopsis* [29]. To identify significant matches, BLASTp was performed with default parameters with an e-value lesser than 0.00001, and the bit score was set to be greater than 100. All the insignificant hits were excluded. The selected sequences were confirmed by screening for the peptidase domain (Peptidase C_14, pfam00656) by subjecting the sequences to Conserved Domains Database (CDD) [30].

The nomenclature of the selected genes was performed on the basis of initials of the rice species. The first letter “O” signifies *Oryza*, second and third letters stand for the species name, for example *OJa, OIn, ORu, ONi, OBa, OBr, OGl, OGb, OPu, OMe*, and *OLo* stands for *O. sativa* subsp. *japonica*, *O. sativa* subsp. *indica*, *O. rufipogon*, *O. nivara*, *O. barthii*, *O*. *brachyantha*, *O. glumaepatula*, *O. glaberrima*, *O*. *punctata*, *O. meridionalis,* and *O. longistaminata,* respectively. MC denotes the metacaspase family, which is followed by a numeral.

### 2.2. Gene Structure, Motif Analysis, and Phylogenetic Tree Construction

The Gff3 (General feature format) files were used to retrieve the information regarding the exons and introns in the metacaspase genes. The gene structure was visualized by Gene Structure Display Server 2 [31]. Similarly, the MEME Suite was used to identify the conserved motifs in the metacaspases protein sequences [32]. Multiple sequence alignment was performed by ClustalW provided in the MEGA (version 10.0) software tool. The phylogenetic tree was constructed for protein sequences of 92 *Oryza* metacaspase genes by the Neighbor-Joining Tree Method with JTT + G + I model with bootstrap value 1000 replicates in Mega X software [33].

### 2.3. Chromosomal Localization, Gene Synteny, and Cis-Regulatory Element Analysis

On the basis of genomic annotations retrieved from EnsemblPlants, the chromosomal locations of metacaspases were visualized by Mapchart2.2 [34] and Circos tool [35]. Along with the chromosomal localization, the relationship between the genes was visualized using the Circos tool. The 2 kb upstream genomic region was retrieved for all the 92 metacaspase genes and subsequently analyzed by PlantCARE and PLACE databases to identify the transcription factor binding elements [36].

### 2.4. Localization and Tertiary (3D) Protein Structure Analyses

Various in silico tools, including CELLO [37], TMHMM 2.0 [38], and ProtParam [39] were used to study the localization of metacaspase proteins. A gene annotation study was conducted to annotate the roles of metacaspase proteins. The protein tertiary structure for the metacaspases was developed using I-TASSER (Iterative Threading ASSEmbly Refinement) server [40].

### 2.5. Haplotypic Evaluation

Haplotypic evaluation of the metacaspase genes was performed based on the sequence variations, including single-nucleotide polymorphisms (SNPs) and InDels. Whole-genome resequencing information available for 4500 diverse rice genotypes provided at RiceVarMap2.0 was used for the haplotyping of the metacaspase genes [41]. A haplotyping network was developed to understand the evolution of different haplotypes in respective rice groups such as *O. sativa* subsp. *indica* and *japonica,* and *tropical*.

### 2.6. Transcriptomic Evaluation and Co-Expression Analysis of Metacaspase Genes

Transcriptomic data (RNA-Seq) were retrieved from the Sequence Read Archive (SRA) at the National Center for Biotechnology Information (NCBI) to study the expression pattern of metacaspase genes. The data related to developmental, abiotic, and biotic stress conditions such as *Magnaporthe oryzae* infection, cold, drought, and salt stress conditions in susceptible and resistant cultivars were downloaded in the fastq format. CLC Genomic Workbench 12 (http://resources.qiagenbioinformatics.com/) was used to obtain the expression values in terms of reads per kilobase of transcripts, per million mapped reads (RPKM). The raw reads were processed and then mapped to the respective genomes with default parameters. The RPKM values were then used to develop the co-expression network by CoExpNetViz. The co-expression network was visualized using Cytoscape 3.7.1. Highly co-expressed genes were highlighted with a larger label size. Furthermore, log2-transformed RPKM expression values were used to generate a heatmap to show the expression pattern in different conditions by performing hierarchical clustering analysis using the Euclidean distance metric and average linkage clustering linkage method implemented in Multiple Experiment Viewer (MeV) v 4.9.0 software.

### 2.7. Plant Materials and Fungal Infection

To study the response of metacaspase genes family in compatible and incompatible interactions, a resistant rice genotype Tetep and a susceptible genotype Pusa Basmati-1 were subjected to infection with *Magnaporthe oryzae* race Pani NB. The plants were grown in a greenhouse under controlled environment conditions (16 h light/8 h dark at 25 °C) for three to four weeks. The *M. oryzae* infection assay was performed as earlier described by Valent et al. [42], with some modifications. At the 3–4 leaf stage, each plant was inoculated with 20–25 mL of blast spore suspension (10^5^ spores/mL). To ensure uniform application of inoculum, an atomizer sprayer was used. The plants were maintained in complete darkness and 100% relative humidity for the initial 24 h. Later, the photoperiod was adjusted to the 16 h/8 h dark, keeping the relative humidity at 70–80%. Sampling was performed at five time-points, 0, 12, 24, 36, and 48 h post-infection in three biological and technical replicates each.

### 2.8. RNA Extraction and Expression Analysis

Total RNA isolation was performed by using Spectrum^TM^ Plant Total RNA Isolation Kit (Sigma Aldrich, St. Louis, MO, USA) following the manufacturer’s protocol. The quality and the quantity of the total isolated RNA was checked on agarose gel electrophoresis and Nanodrop Lite UV–Visible spectrophotometer (Thermo Fisher Scientific, Waltham, MA, USA). Total RNA was used to synthesize cDNA using iScript^TM^ cDNA Synthesis kit (Bio-Rad, Hercules, CA, USA) following the manufacturer protocol. The primers for the real-time quantitative qRT-PCR were designed by QuantPrime online tool (https://quantprime.mpimp-golm.mpg.de/). The qRT-PCR was performed in 10 µL PCR mixture (5 µL SYBRGreen, 0.3 µL forward and reverse primer each, 1 µL cDNA (5 ng/µL), 3.4 µL ddH_2_O). The house-keeping gene *Ubiquitin* was used as a reference to normalize the expression values of target genes. The relative expression calculations were performed using the 2^−ΔΔCt^ formula [43] for three biological and technical replicates each.

## 3. Results

### 3.1. Metacaspases in Wild and Cultivated Rice Genomes

**Figure 1:**
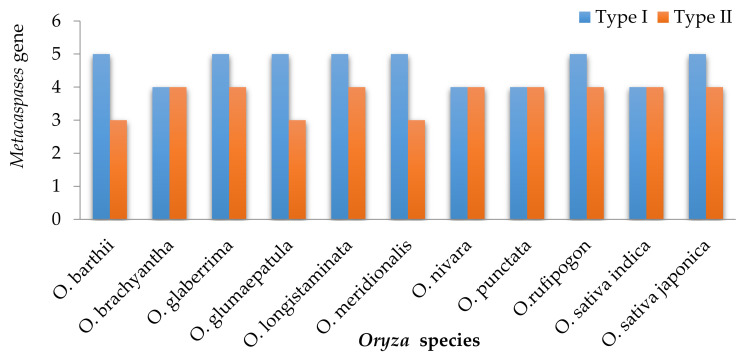
Distribution of the number of metacaspase genes in wild and cultivated *Oryza* species. Blue bars represent Type I metacaspase genes, and red bars represent Type II metacaspase genes.

A homology-based search performed in ten *Oryza* species initially identified 93 metacaspase genes. Furthermore, the peptidase domain (PF00656) was identified in all genes (Supplementary File S1). One of the candidate genes (*OBART11G02150.1*) was omitted from the analysis despite the presence of the peptidase domain, due to the presence of a redundant sequence. The number of metacaspase genes was roughly similar in all ten wild and cultivated *Oryza* species under study (Figure 1). Eight metacaspase genes were found in *O. brachyantha, O. sativa* subsp. *indica, O. glumaepatula, O. barthii, O. meridionalis, O. nivara*, and *O. punctata* genomes and nine in *O. longistaminata, O. sativa* subsp. *japonica, O. glaberrima* and *O. rufipogon* genomes. A single peptidase domain was found to be present in almost all metacaspase genes with the exception of *ONiMC2, OPuMC1,* and *OPuMC2* where two peptidase domains were observed (Supplementary File S1). The metacaspase genes were divided into two types, Type I and Type II on the basis of the length of the linker region between p10 and p20 subunits and the presence of additional domains. An additional 24–26 amino acids long zinc finger domain (*zf*-domain) is present in some Type I metacaspase genes. Contrary to it, a 45–46 amino acid long *zf*-domain was found in *O. rufipogon*. A maximum of two genes with *zf*-domains were found in *O. sativa* subsp. *japonica*, *O. glaberrima*, and *O. rufipogon,* whereas no single *zf*-domain was found in the metacaspase genes from *O. brachyantha, O. glumaepatula, and O. longistaminata*. Only one zf-domain was present in *O. barthii, O. meridionalis, O. nivara, O. punctata*, and *O. sativa* subsp. *indica*.

### 3.2. Predicted Structural and Functional Attributes of Metacaspase Genes

The proteins in this family were found to be highly variable (Supplementary File S2, Table 1). Mostly the metacaspase protein length falls in the range of approximately 200–400 amino acids. The smallest metacaspase protein was found to be 120 amino acids long (OLoMC1); whereas, the largest protein found was 1086 amino acid long (ONiMC8). The average size and molecular weight of all 92 metacaspase genes under study were 404 amino acids and 40 kDa. The pI ranges between 5.24 and 11.59, which indicates both the acidic and basic nature of the protein. Mostly, all the metacaspase proteins are acidic or slightly acidic in nature, but a few among them possess basic characteristics. Similar to the molecular weight, the gravy score fluctuates between −0.713 and −0.008. The studies regarding the subcellular localization of metacaspase proteins in the cell suggested that localization of metacaspase proteins in the cell is also highly variable. Few metacaspase proteins showed the presence of signals for the plasma membrane, whereas a single protein contains the signal sequence for mitochondrial localization (OBrMC1). Gene annotation studies revealed the role of metacaspase proteins majorly in biological processes and molecular functions (Supplementary Figure S1). The three-dimensional structure studied by the iTASSER server revealed the similar architecture of metacaspase proteins with a high level of structural conservations (Supplementary File S3).

### 3.3. Gene Architecture, Conserved Motifs, and Phylogenetic Distribution of Metacaspase Genes

**Figure 2:**
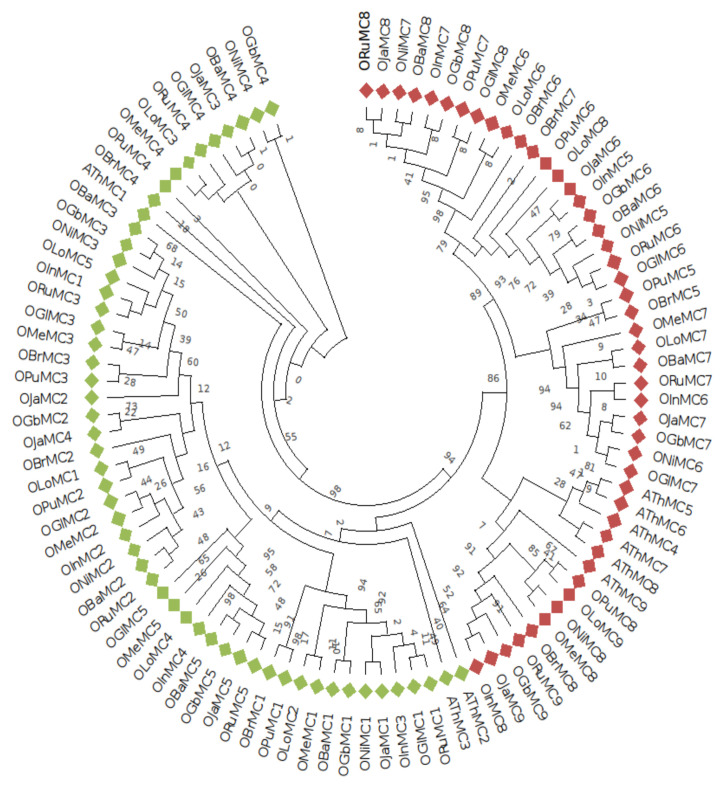
Neighbor-Joining phylogenetic tree of metacaspase genes from wild and cultivated *Oryza* species. The phylogenetic tree comprising 92 *Oryza* and 9 *Arabidopsis* metacaspase genes was generated using Mega X software. Red square tiles represent Type II metacaspase genes whereas green square tiles indicate Type I metacaspases. Gene nomenclature used OJa for *japonica*, OIn for *indica*, ORu for *rufipogon*, ONi for *nivara*, *barthii* is named as OBa, *brachyantha* as OBr, *glumaepatula* as OGl, *glaberrima* as OGb, OPu stands for *punctata*, OMe stands for *meridionalis*, and OLo is for *longistaminata,* and MC denotes the metacaspase family, which is followed by a numeral representing the specific gene.

The evolutionary relationship among the metacaspases identified in the ten *Oryza* species was studied by phylogenetic tree construction. Metacaspase proteins on the basis of their domains were categorized into Type I and Type II groups, which were also grouped into two separate clades in the phylogenetic tree (Figure 2). Out of the entire 92 metacaspases in rice species and 9 in *Arabidopsis* metacaspases under study, 54 and 47 proteins belonged to Type I and Type II, respectively (Figure 2).

**Figure 3:**
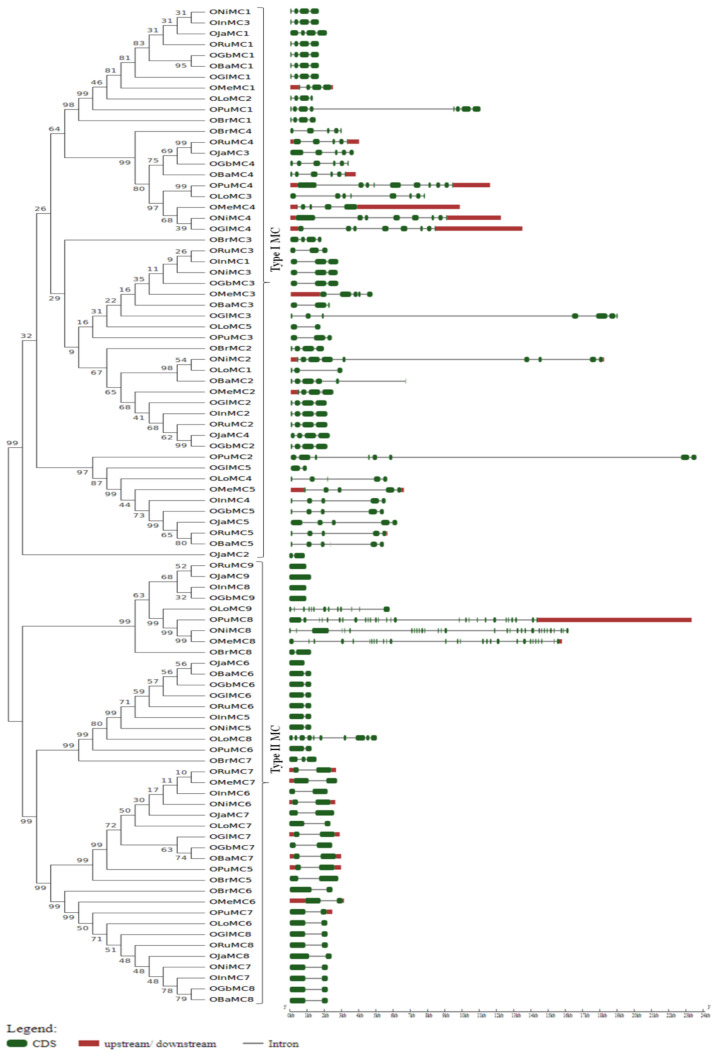
Exon–intron architecture of metacaspases identified in ten *Oryza* species. Exons, introns, and untranslated region are indicated by green, grey, and red, respectively. The coding DNA sequence (CDS) and genomic sequences of metacaspase from all ten *Oryza* species were used to develop pictorial presentation using the GSDS 2.0 online tool. Gene nomenclature used OJa for *japonica*, OIn for *indica*, ORu for *rufipogon*, ONi for *nivara*, *barthii* is named as OBa, *brachyantha* as OBr, *glumaepatula* as OGl, *glaberrima* as OGb, OPu stands for *punctata*, OMe stands for *meridionalis*, and OLo is for *longistaminata,* and MC denotes the metacaspase family, which is followed by a numeral representing the specific gene.

**Figure 4:**
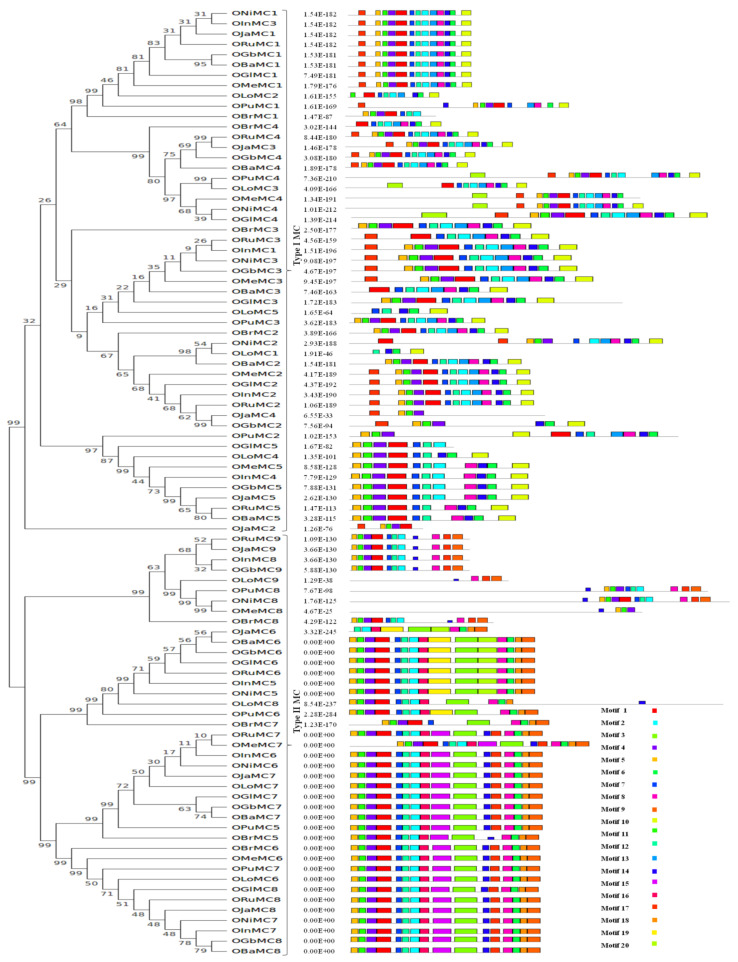
Conserved motifs identified in metacaspase protein from ten *Oryza* species. The motifs were identified by MEME online tool (http://meme-suite.org/) using default parameters. Gene nomenclature used OJa for *O.* *sativa* subsp. *japonica*, OIn for *O.* *sativa* subsp. *indica*, ORu for *rufipogon*, ONi for *nivara*, *barthii* is named as OBa, *brachyantha* as OBr, *glumaepatula* as OGl, *glaberrima* as OGb, OPu stands for *punctata*, OMe stands for *meridionalis*, and OLo is for *longistaminata,* and MC denotes the metacaspase family, which is followed by a numeral representing the specific gene.

A thorough study was conducted to analyze the gene structure and conserved domains present in the metacaspase proteins in the *Oryza* species. An exon–intron organization for all the metacaspase genes in wild and cultivated rice species was constructed to understand the variations in the coding and non-coding metacaspases sequences (Figure 3). The number of introns in the metacaspase genes is highly variable, spanning from as low as one to as high as thirty introns. Exceptionally, *ORuMC9, OJaMC9, OInMC8, OGbMC9, OJaMC6* genes were found to be intron-less, whereas *OPuMC8, ONiMC8, OMeMC8, OLoMC9* genes had more than 25 introns. The genes coding for longer metacaspases possessed a higher number of introns. Interestingly, the protein length of all genes with no intron was 341 amino acids with the exception of *OJaMC6* (305 amino acids). A total of twenty conserved motifs were identified in metacaspase proteins using the MEME software tool (Figure 4). Subsequently, a CDD search was performed to annotate the identified motifs. Interestingly, the domain architecture in Type II metacaspases was very much similar to that in the Type I metacaspases group.

### 3.4. Chromosomal Distribution, Gene Duplication, and Syntenic Relationship of Metacaspase Genes in Wild and Cultivated Oryza Species

**Figure 5:**
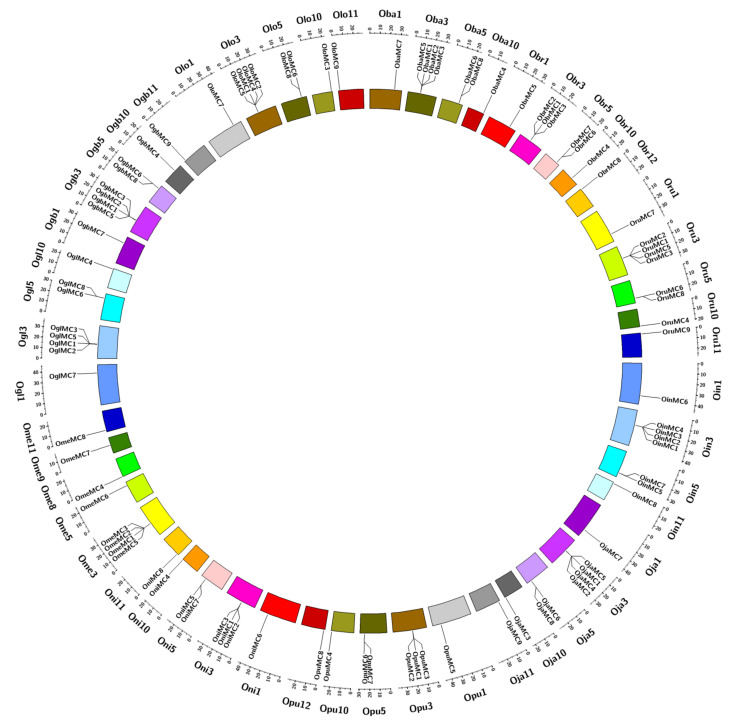
Distribution of metacaspase genes in ten *Oryza* species genomes. Green represents Type I metacaspase genes, and red represents Type II metacaspase genes. The outer circle represents the chromosomes of ten *Oryza* species carrying the metacaspase genes. The inner circle denotes the chromosomal location of all 92 metacaspase genes.

Metacaspase genes were very asymmetrically distributed on eight different chromosomes (Chr.) in *Oryza* species (Figure 5). No metacaspase genes were found to be located on Chr. 2, Chr. 4, Chr. 6, and Chr. 7 in all ten *Oryza* species. Interestingly, 42% of total metacaspase genes, i.e., 39 out of 92, were found to be located on Chr. 3. Gene duplication events were analyzed to understand the duplication event that possibly happened during the evolution of metacaspase genes. The results suggested that metacaspase genes evolved through the segmental and proximal duplication events over tandem and dispersal duplications (Supplementary Table S1). Exceptionally, O. *meridionalis* showed dispersal duplication in all of its genes. The syntenic relation was also studied in order to gain a better understanding of the relationship between the metacaspase genes. As expected, a high level of synteny between metacaspase genes from different *Oryza* species was observed (Supplementary Figure S2).

### 3.5. Cis-Acting Promoter Analysis

**Figure 6:**
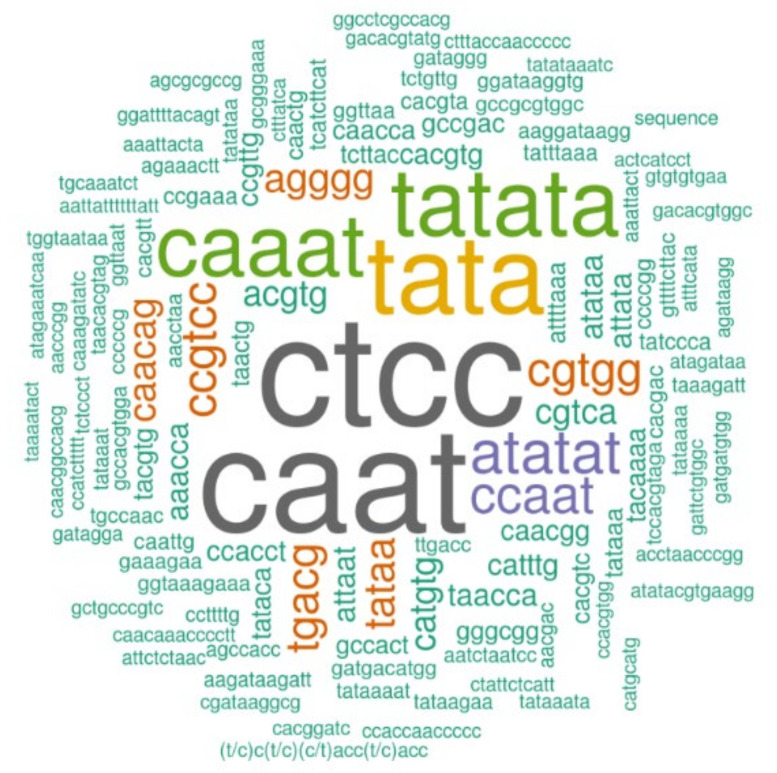
Frequency of transcription factor binding motifs identified in the 2 kb upstream region of the metacaspase genes in ten *Oryza* species. The variation in size represents the abundance of the respective sequences.

Promoter analysis was carried out in metacaspase genes identified in *Oryza* species to gain a better understanding of the gene regulation across the species (Supplementary File S4). Many stress, development, and light-responsive *cis*-regulatory elements were found in 2 kb upstream regions of the metacaspase genes along with the most commonly occurring *cis*-regulatory elements which include CAAT, CTCC, TATA, and TATATA box (Figure 6). Light responsive G-box was present majorly in all the metacaspase genes of *Oryza* species; in contrast, Sp1 (GGGCGG), which is also related to light responsiveness, was found in some metacaspase genes. Identified *cis*-elements known to be participating in abiotic and biotic stresses include WRKY-related (S000447, S000390, and S000457), MYB-related (S000176), and MYC-related (S000407) [44,45]. Abscisic responsive elements known as ABRELATED1 (S000414), ABRERATICAL (S000408), and ABREOSRAB21 (S000012) and a GCC box (S000430) were also found to be located in the upstream regions of the metacaspase genes. Along with this an auxin-responsive element, NtBBF1 (S000273), which was found in a few metacaspase genes [46] (Supplementary File S4). The most frequently occurring stress-related *cis*-elements in all the *Oryza* species were Myc-related MYCCONSENSUSAT and WRKY71OS, whereas in *O. glumaepatula* they were MYCCONSENSUSAT and WBOXNTERF3. Auxin-related *cis*-element, NtBBF1 (S000273), was found in two or three members of each *Oryza* species except for *O. brachyantha* where the NtBBF1 promoter element was present in six metacaspase genes.

### 3.6. Haplotyping for Genes in Rice

Haplotyping of *Oryza* metacaspase genes was performed using whole-genome sequencing information data obtained from RiceVarMap2.0 (Supplementary Files S5 and S6). Consequently, haplotype networks were developed to depict the distribution of haplotypes across *Oryza* subgroups. As shown in Table 2, gene Os03t0389100-01 depicted the maximum number of non-synonymous mutations, whereas the minimum number of non-synonymous mutations was observed in Os10t0565100-00. Similarly, haplotypes based on the number of SNPs were found to be maximum in number for Os03t0389100-01 and minimum for Os05t0496400-00. The effects of amino acid variations detected in the metacaspase genes were further studied using the PROVEAN tool (Supplementary File S7). PROVEAN results revealed no deleterious mutations present in Os01t0799900, Os03t0389501, Os03t0389000, and Os11t0134700, whereas maximum deleterious mutations were present in Os03t389100.

### 3.7. Expression Analysis of Metacaspase Genes during Different Developmental and Stress Conditions

**Figure 7:**
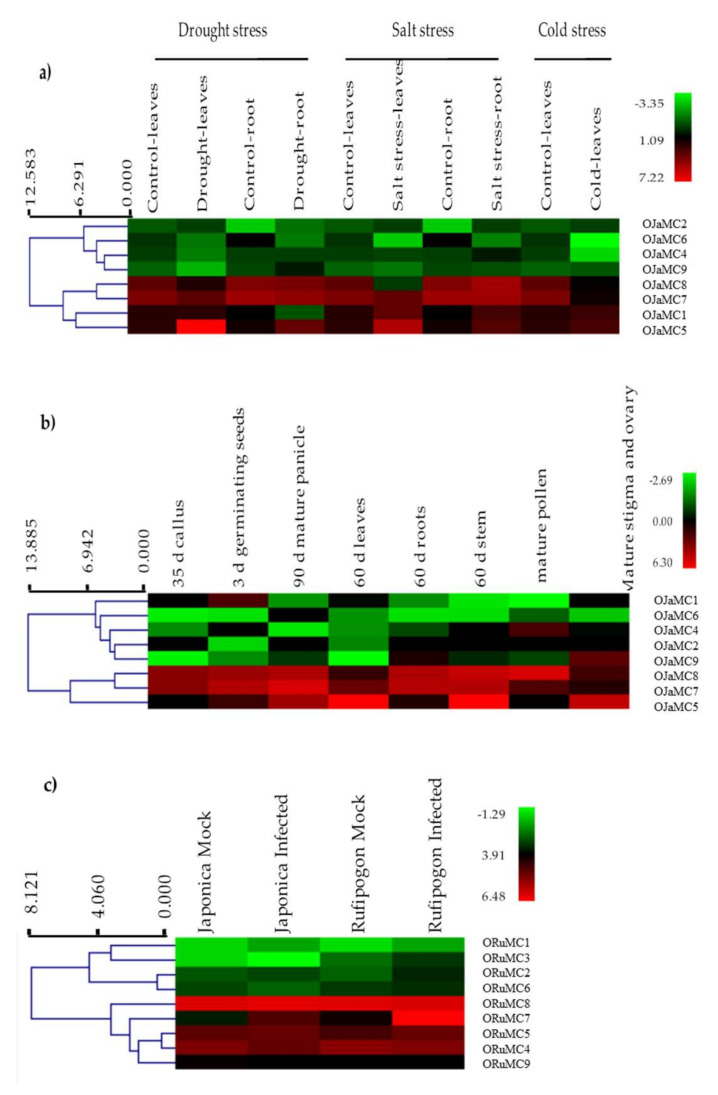
Heatmaps of metacaspase gene expression generated from RNA-Seq datasets under different abiotic stress conditions, different developmental stages and tissues, and biotic stress in rice. (**a**) Expression of metacaspase genes in *O. sativa* subsp. *japonica* under various abiotic stress, (**b**) gene expression analysis for *O. sativa* subsp. *japonica* across different developmental stages in tissues such as callus, germinating seed, panicle, leaves, roots, stem, pollen, stigma, and ovary. (**c**) Hierarchical clustering analysis for *O. sativa* subsp. *japonica* and *O. rufipogon* gene expression data under mock and infection cases.

For a complete expression atlas of metacaspase genes, the RNA-seq data were retrieved from PRJNA134239, which comprises 43 independent RNA-seq libraries for *O. japonica* (cv. *Nipponbare*) tissues. The expression pattern of metacaspase genes was studied for abiotic, biotic, and developmental stages using data from different studies. No expression was observed for *OJaMC3* across different RNA-seq libraries, therefore hierarchical clustering of eight genes across abiotic stress in *O. sativa* was performed. Expression analysis revealed categorization of these genes into two groups, wherein four genes showed moderate-to-low gene expression under drought, salt, and cold conditions (Figure 7a, Supplementary File S8). The *OJaMC5* gene has higher expression in leaves under drought and salt stress compared to that in control conditions. Similarly, *OJaMC8* and *OJaMC7* has higher expression in leaves under control condition compared to that in cold stress (Figure 7a). Gene expression clustering across different developmental stages and tissues of *O. sativa* revealed a similar clustering into two groups (Figure 7b). However, among eight genes, five clustered under a single group and the rest into the second group. Based on expression patterns, the group with five genes showed low-to-moderate gene expression, whereas the remaining group showed moderate-to-high gene expression for several developmental stages. The *OJaMC5* gene showed higher expression in 60 days old leaves, stem, and mature stigma and ovary (Figure 7b).

The comparative expression analysis of metacaspase genes in *Oryza* species was studied using the RNA-seq data retrieved from the bioproject PRJNA393480. The study was conducted on root tissue for *Oryza* species *O. rufipogon* (Dongxiang) and *O. sativa japonica* (Dongdao-4) when exposed to *M. oryzae* (Guy 11) under controlled environmental conditions in triplicates. The expression pattern of all nine metacaspase genes was studied in all control and test samples. The *ORuMC*7 gene was found to have high expression in both *O. sativa* subsp. *japonica* and *rufipogon* samples as compared to control, but the RNA-seq expression of *ORuMC*7 was much higher in wild species as compared to that in cultivated ones (Figure 7c).

**Figure 8:**
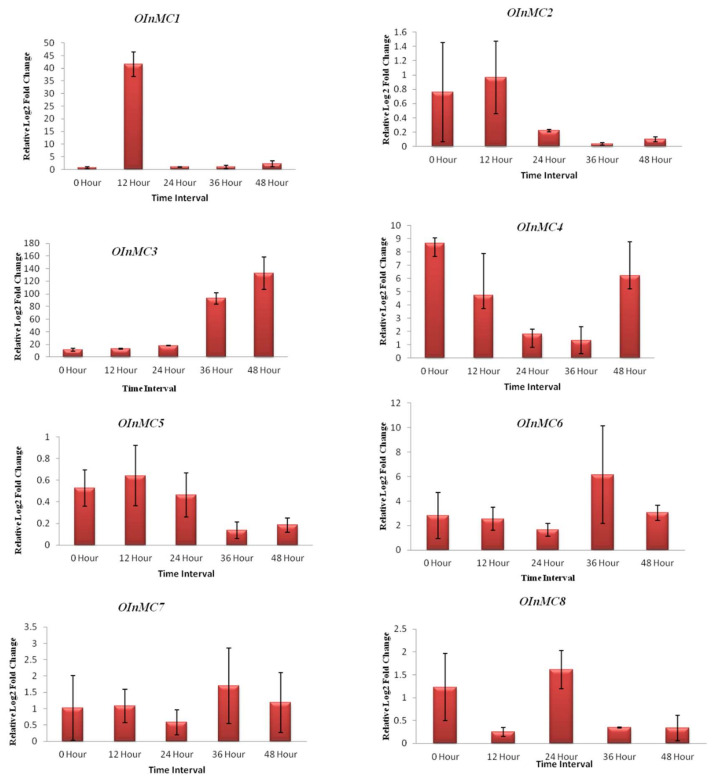
Relative log 2-fold change of the eight (*OInMC1-8*) genes with response to biotic stress posed by *Magnaporthe oryzae* at different time intervals after inoculation on rice cultivar Pusa Basmati 1. The house-keeping gene *Ubiquitin* was used as a reference to normalize the expression values of target genes. The relative expression calculations were performed using the 2^−ΔΔCt^ formula [43] for three biological and technical replicates each.

The expression profiling of metacaspase genes against blast disease in the *O. sativa indica* resistant and susceptible varieties, Tetep and Pusa Basmati-1, respectively, were carried out in controlled lab conditions. The expression of all eight *OInMC*(1-8) genes was studied by qRT-PCR (Figure 8). The relative expression values of all of the *OInMC* genes were found to be increasing at different time intervals. A high relative log2 fold change value was obtained for *OInMC1* and *OInMC3* genes 12 and 36 h post-infection (hpi), respectively.

## 4. Discussion

The metacaspase genes have been studied in several plant species to understand their role in stress and developmental phases of the growth cycle [29,47]. The number of metacaspases in plant species varies considerably, for instance, 6 in grapes, 8 in rice and tomato, 9 in *Arabidopsis*, and 30 in cotton species [18]. Such wide range of occurrence may be due to the high level of variation in duplicated genome and ploidy level in plants. The genome-wide study of metacaspases in the *Oryza* clade will help to better understand its functional role and evolution in rice. The in-depth understanding of metacaspases in wild relatives will also provide an opportunity to introduce desirable alleles from wild genetic resources. Here, identification, characterization, and distribution of metacaspase genes in ten *Oryza* species were performed, which uniformly identified 8 or 9 homologs in each species despite having differences in the origin of the genomes (AA, BB, or FF) (Figure 1, Table 1). Recently, genome-wide identification of metacaspase genes performed in four *Gossypium* species identified a varying number of homologs: 16 in *G. raimondii*, 17 in *G. arboreum*, 26 in *G. hirsutum*, and the highest, 30, in *G. barbadense* [48]. Such a wide variation among *Gossypium* species as compared to the *Oryza* species is mostly due to the higher ploidy level and proportion of the duplicated genome.

The metacaspase genes were classified into Type I and Type II metacaspases on the basis of the linker region between p10 and p20 domains (Figure 2, Supplementary File S1). An additional domain, zinc finger (*zf*) domain, is present in the structure of some of the Type I metacaspase genes. However, the *zf* domains were completely absent in the Type I metacaspase genes of *O. brachyantha, O. glaberrima*, and *O. longistaminata*. In contrast, a single *zf* domain was found in Type I metacaspase genes of *O. barthii, O. meridionalis, O. nivara, O. punctata,* and *O. sativa indica*. Two genes with *zf* domains were present in *O. sativa japonica*, *O. glaberrima,* and *O. rufipogon*. It is very surprising to see present/absent variation for the *zf*-domain among *O. sativa* subsp. *indica* and *japonica* since both subspecies belong to *O. sativa.* The *zf* domains are thought to be involved in protein–protein interactions. A study performed in yeast has shown active involvement of *zf* domains in the interaction of AtMC1 with LSD-1 in the case of the *Oryza* genus [49]. Therefore, the present/absent variation for the *zf* domains amongst species will help to study the significance of this domain.

Information about the exon–intron organization of metacaspase genes helps to predict the origin, expression activity, as well as possibilities of alternate splicing [50]. The number of introns was higher in Type I metacaspase genes as compared to that in Type II MC (Figure 3). The number of introns was comparatively very high in *O. longistaminata, O. punctata, O. nivara, O. meridionalis* wild species, whereas three or four introns and intron-less metacaspase genes are common in *O. sativa* (*japonica* and *indica*)*, O. rufipogon, O. barthii*, and *O. brachyantha*. The exon–intron organization study was consistent with the earlier reported studies about the number of introns higher in Type I than in Type II metacaspases [13,51]. Gene duplication plays an important role in the loss of introns. In general, recently evolved genes tend to have fewer introns. However, this is very controversial to say whether intron loss is dominant in higher plant or intron gain [50]. Nevertheless studies in rice clearly indicated that the newly evolved genes tend to have fewer introns and have more chances to lose their expression during the evolution [50]. Similarly, the organization of conserved motif also helps to understand the functional domains as well as the evolutionary relationship of the metacaspase gene family members. Here, a total of 20 conserved motifs identified with the MEME tool (Figure 4), including the signature p10 and p20 domains, and the overall organization looked highly conserved in the two types of metacaspases.

In the present study, whole-genome resequencing data from 4726 diverse genotypes were accessed to get the sequence variation in metacaspase genes. The sequence analysis helped to locate all the SNPs and InDels and subsequently predicted their effect on protein functionality (Table 2). The deleterious mutations identified in the present study will be helpful in understanding the role of metacaspases. Earlier, such deleterious and neutral variation predicted with the PROVEAN tool was successfully validated using a site-directed mutagenesis experiment performed with *SlNIP2-1*, *PtNIP2-1*, and *OsNIP2-1*, respectively, from tomato, poplar, and rice [52]. Such predicted deleterious variation in the metacaspase genes need to be experimentally validated. The publicly available whole-genome resequencing data provide an opportunity to understand the functional role of metacaspase genes. The sequencing data were also used to define the haplotypes, which will be helpful to develop haplotype-specific markers and also to understand the haplotypic evolution (Supplementary File S5). The haplotypes representing allelic variation existing in the rice germplasm allow us to study the effect of each allele. The variations located in conserved domains can also help to elucidate the molecular mechanism involved in metacaspase activity.

Studying gene expression is essential to understand the tissue-specific or stress-specific function of metacaspase gene family members. Numerous studies have shown stress-induced expression of several metacaspase genes in different plants [47,48,53]. In the present study, available RNAseq transcriptome profiling was used to identify tissue- and stage-specific, stress-specific as well as differential expression among different *Oryza* species of metacaspases genes (Figure 7). The RNA-seq transcriptome data of control and different stress conditions clearly showed that the some metacaspase genes highly expressed in control also had high expression throughout all the stress conditions. Such abundant expression of some metacaspase genes is surprising. In this regard, a recent study where the crystal structure of *Arabidopsis* metacaspase 4 (AtMC4) was evaluated seems helpful to explain why some metacaspases have ubiquitous expression [54]. The structural analysis of AtMC4 revealed activation of the metacaspases only in the presence of Ca^2+^ [54]. Such a Ca^2+^-dependency explains several phenomena that were elusive before. The study suggests that the highly expressed metacaspase genes are non-functional until an adequate supply of Ca^2+^ occurs. This helps the plant to respond quickly to stress conditions. For instance, Ca^2+^ stored in different cellular compartments get fluxed in the cytoplasm with stress signals, and then Ca^2+^-dependent activation of metacaspase takes place. The constitutive high expression of some of the metacaspases will be surely helpful in Ca^2+^-dependent stress response mechanism. Such a phenomenon may be dominantly observed with metacaspase, but many of the metacaspases family members may have a very specific mode of action. To understand such a tissue- and condition-specific role of metacaspases, more extensive studies using integrated omics approaches are needed.

In addition to extensive RNAseq transcriptome analysis, qRT-PCR analysis of metacaspase genes under *M. oryzae* infection was performed in the present study (Figure 8). The metacaspase genes were found to be differentially expressed after *M. oryzae* infection, but the response time for each of the genes was unique. For instance, *OInMC1* showed upregulation at 12 hpi, *OInMC8* at 24 hpi, whereas *OInMC4* showed upregulation at 48 hpi. This suggests the possible role of all the metacaspase genes in *M. oryzae* resistance mechanism, but they may have a different role in different cellular compartments or at different steps of the stress–response cascade. Similar results have been reported in rubber tree (*Hevea brasiliensis*) where most of the metacaspase genes showed differential expression patterns under various stresses [55]. For instance, eight out of nine metacaspase genes were found to be differentially expressed under drought conditions in rubber trees [55].

## 5. Conclusions

The role of metacaspase genes under stress and developmental processes in plants has been demonstrated in several studies. However, precise molecular mechanisms involving metacaspase genes are not yet well understood. In the present study, 92 metacaspase genes were identified in nine *Oryza* species and two subspecies of cultivated species *O. sativa*. Extensive gene characterization performed in this study will help to explain the functional role of metacaspases. The phylogenetic analysis performed here helped to define the distribution of Type 1 and Type 2 metacaspases in the *Oryza* clade. The distribution of Type 1 and Type 2 across the *Oryza* species is uniform despite having different genomes (AA, BB, or FF). The sequence variations identified in the metacaspase genes will be helpful for the understanding of structural features of the protein, role of conserved domains, allelic effects, and overall functionality of metacaspase genes. The defined haplotypes for metacaspase genes will be helpful to develop markers for breeding applications as well as for genetic studies. In addition, the present study has provided information about the metacaspase gene expression under biotic and abiotic stress conditions, in different tissues and developmental conditions. Metacaspase gene expression was also evaluated with a real-time qPCR assay, which suggested its role in different stages of disease development. The stepwise upregulation and downregulation of different metacaspases indicate the possibility of a metacaspase-regulated cascade of systematic stress response. The detailed information of evolution, distribution, expression, and allelic variation provided here will be helpful to better understand the metacaspases in rice and for the exploration of knowledge in crop improvement programs.

## Figures and Tables

**Table 1:** Details of 92 metacaspase genes identified in eight *Oryza* wild species and two subspecies *indica* and *japonica* of cultivated rice (*Oryza sativa*).

Sr. No.	Selected Genes	MC Type	Gene	Length (aa)	MW (Kd)	pI	Protein Localization
1	OBART01G33990.1	II	*OBaMC7*	417	45.8	5.52	Nuclear
2	OBART03G19910.1	I	*OBaMC1*	368	39.6	7.85	Nuclear and Extracellular
3	OBART03G19930.1	I	*OBaMC2*	372	40.0	5.73	Chloroplast
4	OBART03G19940.1	I	*OBaMC5*	280	30.9	6.12	Nuclear
5	OBART03G19960.1	I	*OBaMC3*	255	27.5	4.95	Extracellular and Plasma membrane
6	OBART05G21540.1	II	*OBaMC6*	409	44.5	6.53	Nuclear
7	OBART05G21550.1	II	*OBaMC8*	420	46.0	6.04	Nuclear
8	OBART10G18850.1	I	*OBaMC4*	334	36.1	6.16	Extracellular
9	OB01G42780.1	II	*OBrMC5*	417	45.8	5.46	Nuclear
10	OB03G29120.1	I	*OBrMC1*	247	27.0	7.6	Nuclear and Mitochondrial
11	OB03G29130.1	I	*OBrMC2*	344	36.7	5.62	Chloroplast and Nuclear
12	OB03G29140.1	I	*OBrMC3*	294	32.0	5.44	Extracellular and Plasma membrane
13	OB05G29040.1	II	*OBrMC7*	440	47.9	8.86	Nuclear
14	OB05G29050.1	II	*OBrMC6*	419	45.8	6.48	Nuclear
15	OB10G25980.1	I	*OBrMC4*	262	28.4	5.15	Extracellular
16	OB12G12140.1	II	*OBrMC8*	409	42.2	4.79	Cytoplasmic
17	ORGLA01G0279200.1	II	*OGbMC7*	417	45.8	5.52	Nuclear
18	ORGLA03G0186200.1	I	*OGbMC1*	368	39.6	7.85	Extracellular, Nuclear
19	ORGLA03G0186300.1	I	*OGbMC2*	397	42.7	11.24	Nuclear
20	ORGLA03G0186400.1	I	*OGbMC5*	302	33.5	6.46	Nuclear
21	ORGLA03G0186500.1	I	*OGbMC3*	369	39.1	7.91	Chloroplast and Plasma Membrane
22	ORGLA05G0187100.1	II	*OGbMC6*	409	44.5	6.53	Nuclear
23	ORGLA05G0187200.1	II	*OGbMC8*	420	46.0	6.04	Nuclear
24	ORGLA10G0139900.1	I	*OGbMC4*	355	38.5	5.8	Extracellular
25	ORGLA11G0018800.1	II	*OGbMC9*	341	35.6	5.09	Cytoplasmic
26	OGLUM01G38250.1	II	*OGlMC7*	417	45.8	5.37	Nuclear
27	OGLUM03G20520.1	I	*OGlMC1*	368	39.7	7.85	Nuclear
28	OGLUM03G20560.1	I	*OGlMC2*	392	41.4	7.91	Chloroplast
29	OGLUM03G20570.1	I	*OGlMC5*	176	19.7	4.98	Cytoplasmic
30	OGLUM03G20600.1	I	*OGlMC3*	443	48.0	8.67	Chloroplast
31	OGLUM05G22740.1	II	*OGlMC6*	409	44.5	6.35	Nuclear
32	OGLUM05G22750.1	II	*OGlMC8*	416	45.5	6.03	Nuclear
33	OGLUM10G18910.1	I	*OGlMC4*	582	63.1	7.91	Extracellular
34	AMDW01032852.1_FGP001	I	*OLoMC1*	120	12.6	4.35	Cytoplasmic, Nuclear
35	KN538687.1_FGP194	II	*OLoMC8*	823	87.3	5.32	Chloroplast
36	KN538687.1_FGP207	II	*OLoMC6*	420	46.0	6.04	Nuclear
37	KN538892.1_FGP005	I	*OLoMC4*	235	26.0	5.46	Nuclear
38	KN538892.1_FGP007	I	*OLoMC2*	272	29.8	5.16	Nuclear
39	KN539149.1_FGP003	I	*OLoMC3*	496	54.4	6.81	Extracellular and Nuclear
40	KN539612.1_FGP001	II	*OLoMC9*	452	49.2	6.63	Plasma Membrane
41	KN540137.1_FGP001	I	*OLoMC5*	157	16.7	4.77	Plasma Membrane, Chloroplast
42	KN540619.1_FGP006	II	*OLoMC7*	417	45.7	5.44	Nuclear
43	OMERI03G17880.1	I	*OMeMC1*	370	39.7	8.16	Nuclear
44	OMERI03G17890.1	I	*OMeMC2*	391	41.3	7.91	Chloroplast
45	OMERI03G17900.1	I	*OMeMC5*	303	33.4	5.83	Nuclear
46	OMERI03G17920.1	I	*OMeMC3*	395	42.0	8.87	Plasma Membrane and Chloroplast
47	OMERI05G19430.1	II	*OMeMC6*	420	45.9	5.94	Nuclear
48	OMERI08G00110.2	I	*OMeMC4*	450	49.0	8.59	Extracellular and Nuclear
49	OMERI09G06400.1	II	*OMeMC7*	518	56.8	6.81	Nuclear
50	OMERI11G02500.2	II	*OMeMC8*	833	90.9	8.27	Plasma Membrane
51	ONIVA01G38790.1	II	*ONiMC6*	417	45.8	5.44	Nuclear
52	ONIVA03G21600.1	I	*ONiMC1*	368	39.6	7.85	Nuclear and Extracellular
53	ONIVA03G21620.1	I	*ONiMC2*	679	73.0	6.77	Chloroplast and Nuclear
54	ONIVA03G21660.1	I	*ONiMC3*	360	38.3	7.5	Plasma Membrane
55	ONIVA05G22210.1	II	*ONiMC5*	409	44.5	6.57	Nuclear
56	ONIVA05G22220.1	II	*ONiMC7*	420	46.0	6.04	Nuclear
57	ONIVA10G21340.1	I	*ONiMC4*	814	87.8	9.05	Extracellular and Nuclear
58	ONIVA11G02360.1	II	*ONiMC8*	1086	117.6	6.95	Plasma Membrane
59	OPUNC01G33030.1	II	*OPuMC5*	417	45.9	5.37	Nuclear
60	OPUNC03G18760.1	I	*OPuMC1*	660	71.9	6.78	Plasma Membrane and Nuclear
61	OPUNC03G18780.1	I	*OPuMC2*	555	60.6	6.1	Cytoplasmic and Nuclear
62	OPUNC03G18790.1	I	*OPuMC3*	294	32.0	5.61	Plasma membrane and Chloroplast
63	OPUNC05G19180.1	II	*OPuMC6*	416	44.3	5.46	Nuclear
64	OPUNC05G19190.1	II	*OPuMC7*	419	46.0	6.14	Nuclear
65	OPUNC10G17260.1	I	*OPuMC4*	968	105.6	9.13	Extracellular, Plasma membrane, and Nuclear
66	OPUNC12G01940.1	II	*OPuMC8*	1019	109.9	6.54	Plasma Membrane
67	ORUFI01G37300.1	II	*ORuMC7*	417	45.8	5.54	Nuclear
68	ORUFI03G20550.1	I	*ORuMC1*	368	39.6	7.85	Extracellular
69	ORUFI03G20570.1	I	*ORuMC2*	400	42.1	7.01	Chloroplast
70	ORUFI03G20590.1	I	*ORuMC5*	268	29.5	5.75	Nuclear
71	ORUFI03G20620.1	I	*ORuMC3*	323	33.7	6.24	Plasma Membrane
72	ORUFI05G22800.1	II	*ORuMC6*	409	44.5	6.35	Nuclear
73	ORUFI05G22810.1	II	*ORuMC8*	420	46.0	6.04	Nuclear
74	ORUFI10G20040.1	I	*ORuMC4*	363	38.8	6.21	Extracellular
75	ORUFI11G02080.1	II	*ORuMC9*	341	35.7	5.32	Cytoplasmic
76	BGIOSGA004618-PA	II	*OInMC6*	417	45.7	5.36	Nuclear
77	BGIOSGA010607-PA	I	*OInMC1*	369	39.0	7.5	Plasma Membrane
78	BGIOSGA010608-PA	I	*OInMC4*	301	33.6	6.46	Nuclear
79	BGIOSGA010610-PA	I	*OInMC2*	400	42.1	7.91	Chloroplast
80	BGIOSGA010611-PA	I	*OInMC3*	368	39.6	7.85	Nuclear & Extracellular
81	BGIOSGA017791-PA	II	*OInMC7*	420	46.0	6.04	Nuclear
82	BGIOSGA017792-PA	II	*OInMC5*	409	44.5	6.35	Nuclear
83	BGIOSGA034558-PA	II	*OInMC8*	341	35.6	5.24	Cytoplasmic
84	Os01t0799900-01	II	*OJaMC7*	417	45.8	5.51	Nuclear
85	Os03t0388900-01	I	*OJaMC1*	368	39.6	7.85	Extracellular and Nuclear
86	Os03t0389000-00	I	*OJaMC4*	424	45.2	11.59	Nuclear
87	Os03t0389100-01	I	*OJaMC5*	302	33.5	6.46	Nuclear
88	Os03t0389501-00	I	*OJaMC2*	208	21.9	6.94	Chloroplast
89	Os05t0496400-00	II	*OJaMC6*	305	33.2	8.46	Nuclear
90	Os05t0496500-01	II	*OJaMC8*	420	46.0	6.04	Nuclear
91	Os10t0565100-00	I	*OJaMC3*	457	49.8	8.47	Nuclear
92	Os11t0134700-01	II	*OJaMC9*	341	35.6	5.24	Cytoplasmic

**Table 2:** Details of haplotypes, single-nucleotide polymorphism (SNP), and the effect of SNP variations predicted in metacaspase genes.

Sr. No.	Gene ID	Gene Nomenclature	Chr.	Chr. Position	Haplotypes	SNPs/InDel	Non-Syn. SNP	Deleterious Mut.	Gene Diversity	
									Pi/BP	Theta/BP
1	Os03t388900	*OJaMC1*	3	15530630–15532454	8	15	7	1	0.15	0.10
2	Os03t389501	*OJaMC2*	3	15586221–15587152	8	11	5	0	0.31	0.11
3	Os03t0389000	*OJaMC4*	3	15563374–15564927	6	16	2	0	0.14	0.11
4	Os05t0496400	*OJaMC6*	5	24383180–24384097	3	8	4	2	0.05	0.12
5	Os05t0496500	*OJaMC8*	5	24384902–24387337	4	8	3	1	0.01	0.12
6	Os01t799900	*OJaMC7*	1	33869142–33871835	10	31	3	0	0.06	0.11
7	Os11t0134700	*OJaMC9*	11	1616990–1618300	9	22	14	0	0.14	0.11
8	Os03t0389100	*OJaMC5*	3	15567899–15572135	128	372	49	5	0.16	0.11
9	Os10t0565100	*OJaMC3*	10	22320808–22323911	5	25	0	NA		

Chr.—chromosome; non-syn.—non-synonymous; SNP—single-nucleotide polymorphism; InDel—insertion/deletion; Mut.—mutation.

## Supplementary Materials

Supplementary materials can be found at https://www.mdpi.com/2073-4425/11/12/1412/s1. Supplementary Table S1. Details of gene duplication types predicted for the 92 metacaspase genes identified in *Oryza* species. Supplementary Figure S1. Functional annotation-based distribution of 92 metacaspase genes identified in *Oryza* species. Supplementary Figure S2. Syntenic relationship between metacaspase genes. The circle represents the *Oryza* chromosomes with metacaspase genes, and the inner network shows their relationship. Supplementary File S1. Details of conserved domains and motifs identified in rice metacaspase genes. Supplementary File S2. Details of metacaspase genes identified in eight *Oryza* species along with two subspecies *indica* and *japonica* of cultivated rice (*Oryza sativa*). Supplementary File S3. Protein tertiary structure of metacaspases proteins identified in *Oryza* species. The structures were constructed by iTASSER server. Supplementary File S4. Details of transcription factor binding domains identified in the 2 kb upstream region of the 92 metacaspase genes in *Oryza* species. Supplementary File S5. Details of haplotypes identified in the metacaspase genes in rice. The haplotypes were identified using whole-genome resequencing data from 4726 genotypes available at Rice Variation Map v2.0 (RiceVar Map 2.0). Supplementary File S6. Distribution and network of haplotypes identified for the metacaspase genes in rice genome. The haplotypes were identified using whole-genome resequencing data from 4726 genotypes available at Rice Variation Map v2.0 (RiceVar Map 2.0). Supplementary File S7. Predictions of SNPs having higher functional impacts using the PROVEAN tool (http://provean.jcvi.org/index.php). Supplementary File S8. Gene expression of *OsJaMCs* genes under different stress conditions constructed by Bio-Analytic Resource for Plant Biology (http://bar.utoronto.ca).
