# Supplementary material for: Evolutionary Understanding of Metacaspase Genes in Cultivated and Wild Oryza Species and Its Role in Disease Resistance Mechanism in Rice
Source: Genes (Basel). 2020 Nov 26;11(12):1412. doi: 10.3390/genes11121412 (PMC7760854; doi:10.3390/genes11121412)
Supplement: Supplementary file 1 [file genes-11-01412-s001.zip › Supplementary Fig 2.pdf]

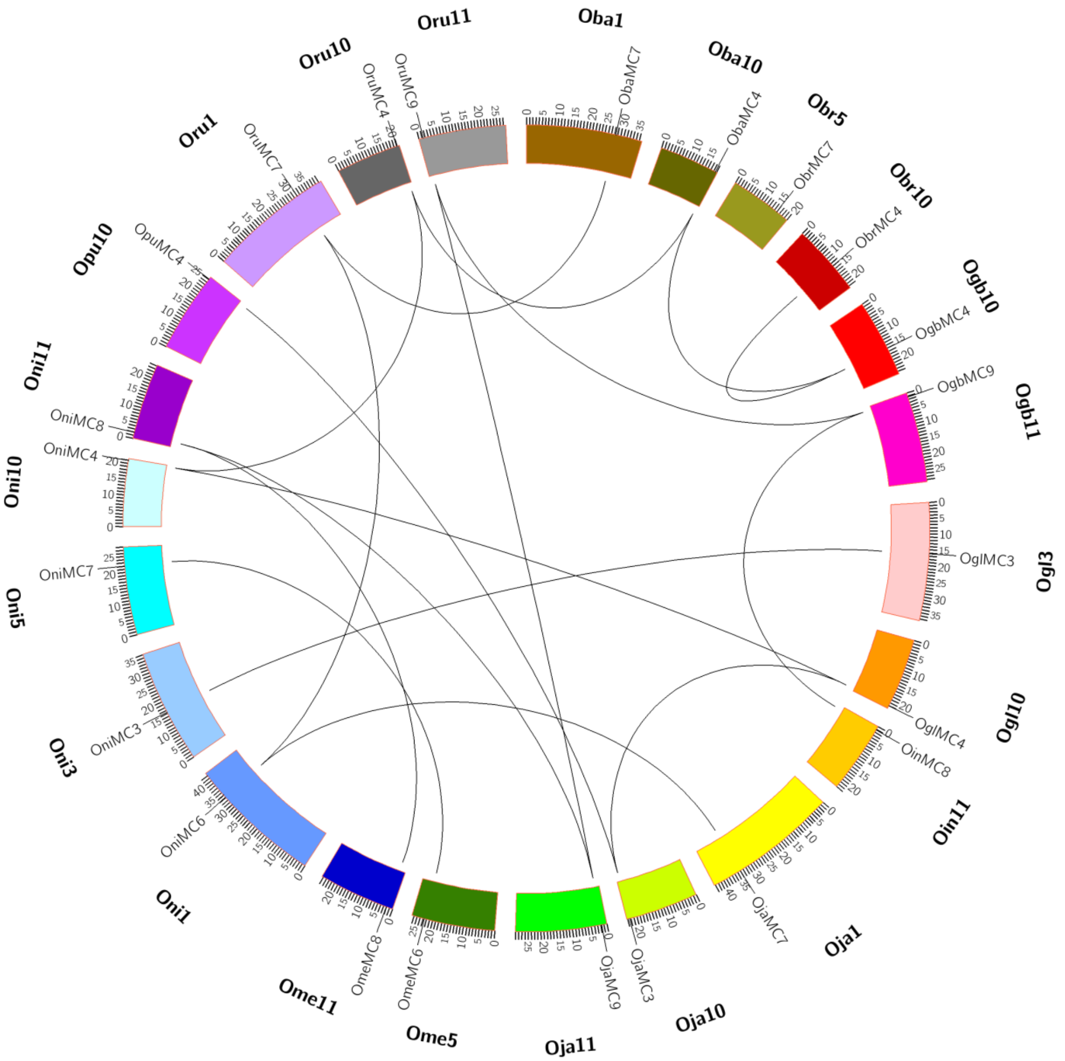

Supplementary Fig. 3. Syntenic relationship between metacaspases genes. The circle represents the *Oryza* chromosomes with metacaspases genes and the inner network shows their relationship.
