# Supplementary material for: Evolutionary Understanding of Metacaspase Genes in Cultivated and Wild Oryza Species and Its Role in Disease Resistance Mechanism in Rice
Source: Genes (Basel). 2020 Nov 26;11(12):1412. doi: 10.3390/genes11121412 (PMC7760854; doi:10.3390/genes11121412)
Supplement: Supplementary file 1 [file genes-11-01412-s001.zip › Supplementary File 3.docx]

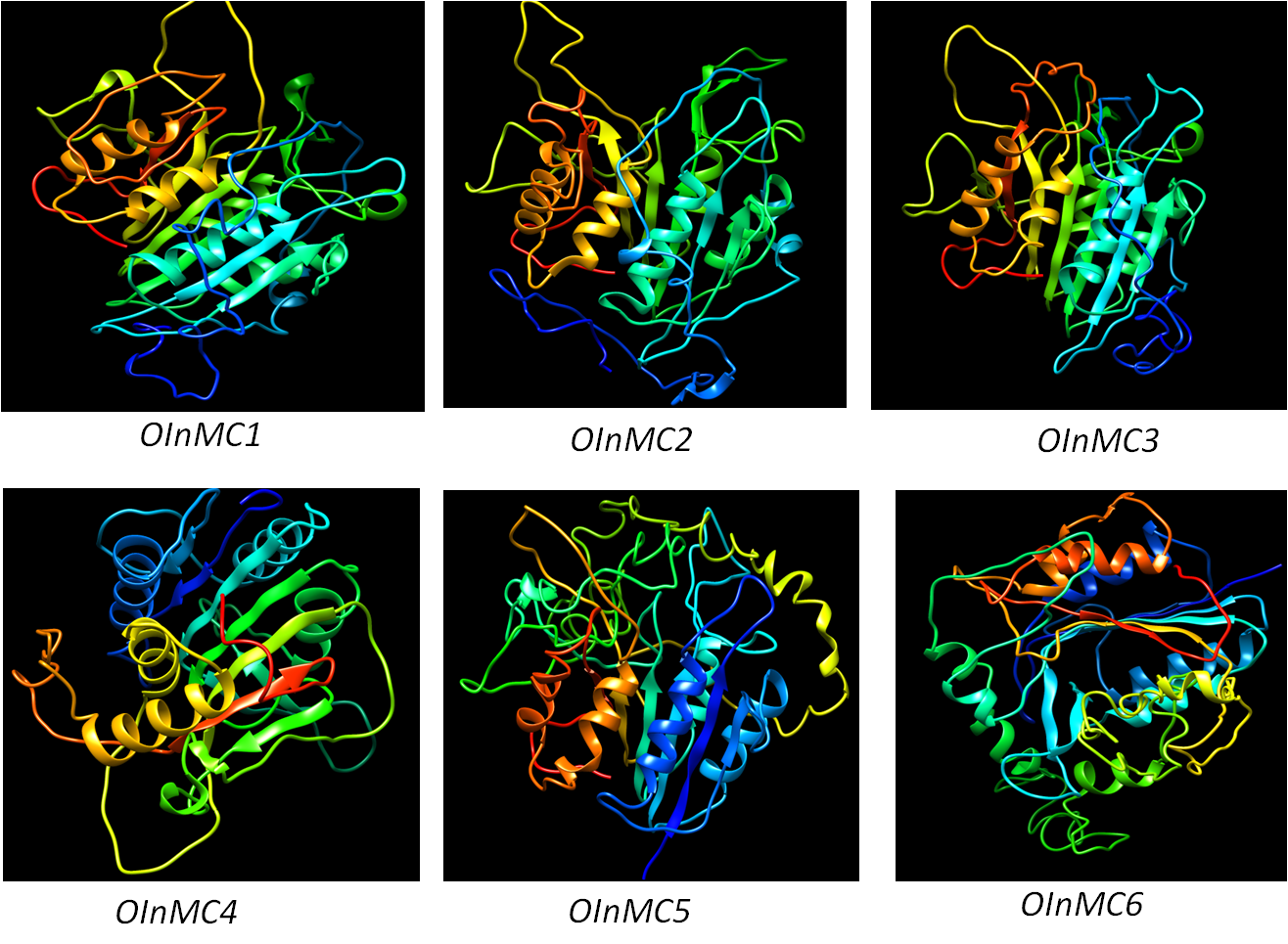


**Protein tertiary structure of metacaspases proteins identified in *Oryza sativa* subspecies *indica*. The structures were constructed by iTASSER server.**


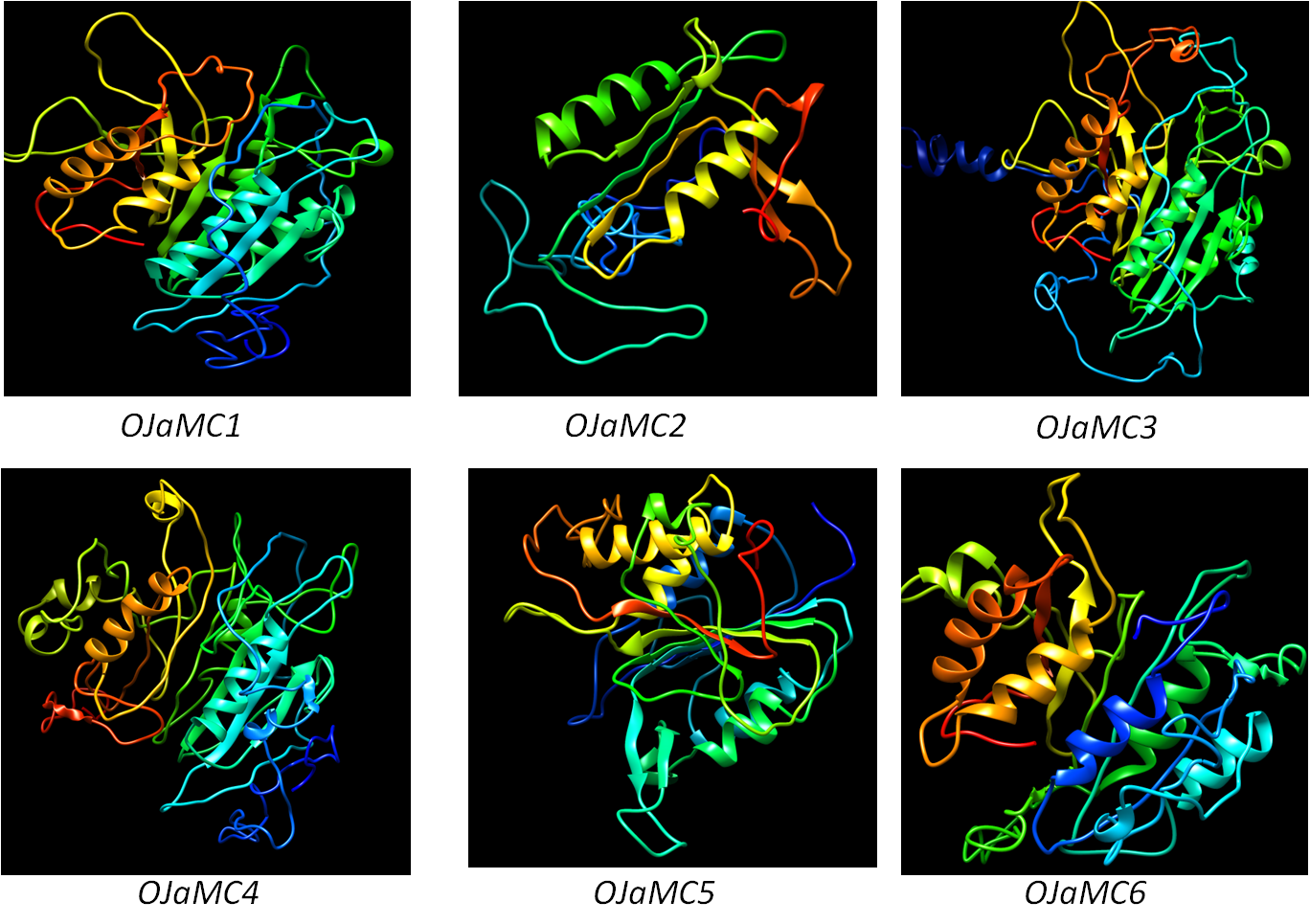


**Protein tertiary structure of metacaspases proteins identified in *Oryza sativa* subspecies *japonica*. The structures were constructed by iTASSER server.**

**Protein tertiary structure of metacaspases proteins identified in *Oryza rufipogon*. The structures were constructed by iTASSER server.**

**Protein tertiary structure of metacaspases proteins identified in *Oryza punctata*. The structures were constructed by iTASSER server.**
