# Supplementary material for: Evolutionary Understanding of Metacaspase Genes in Cultivated and Wild Oryza Species and Its Role in Disease Resistance Mechanism in Rice
Source: Genes (Basel). 2020 Nov 26;11(12):1412. doi: 10.3390/genes11121412 (PMC7760854; doi:10.3390/genes11121412)
Supplement: Supplementary file 1 [file genes-11-01412-s001.zip › Supplementary File 6.docx]

**Haplotyping study for *O.japonica* genes**

- **Os03t388900-01 (*OsJaMC1*)**


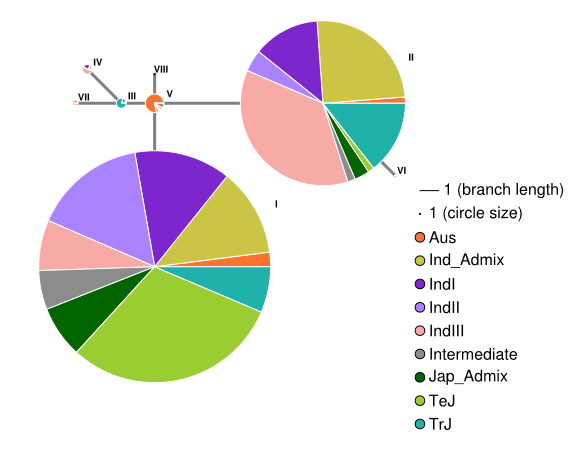


- **Os03t0389501 (*OsJaMC2*)**


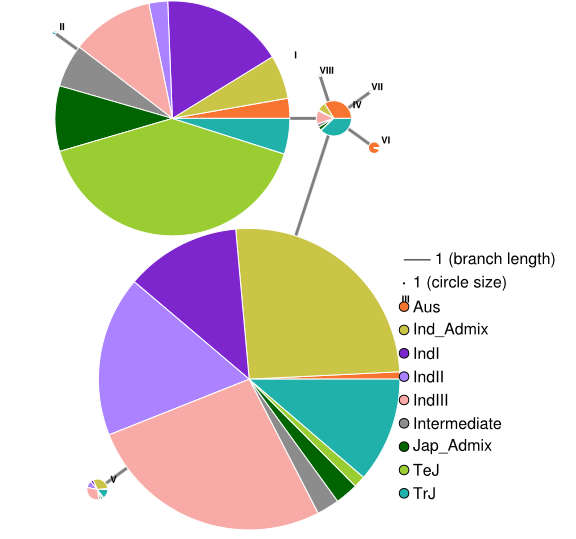


- **Os10t565100 (*OsJaMC3)***

**
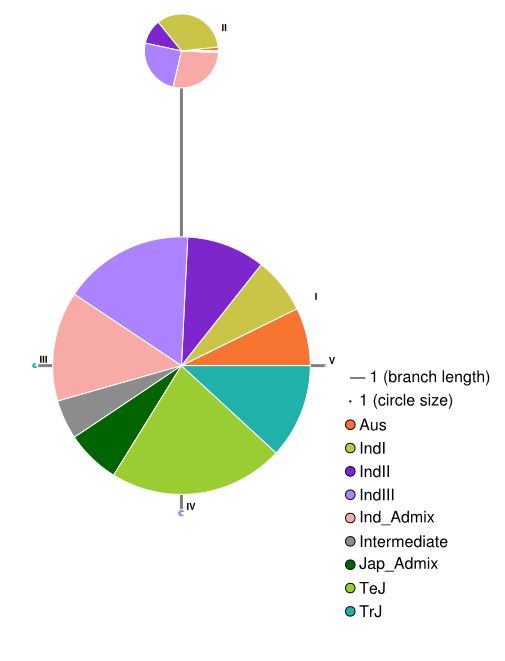
**

- **Os03t0389000 (*OsJaMC4*)**


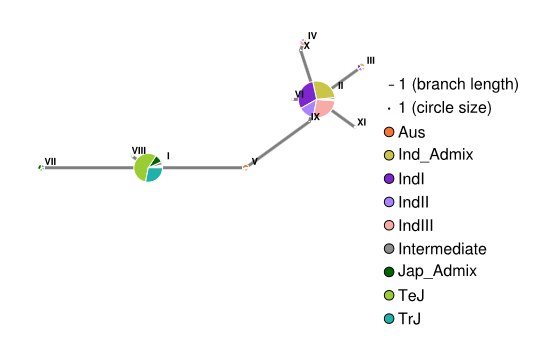


- **Os03t0389100 (*OsJaMC5)***

**
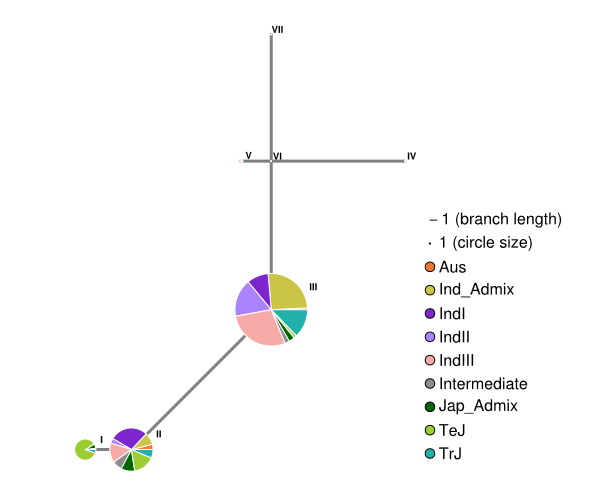
**

- **Os05t0496400 (*OsJaMC6*)**
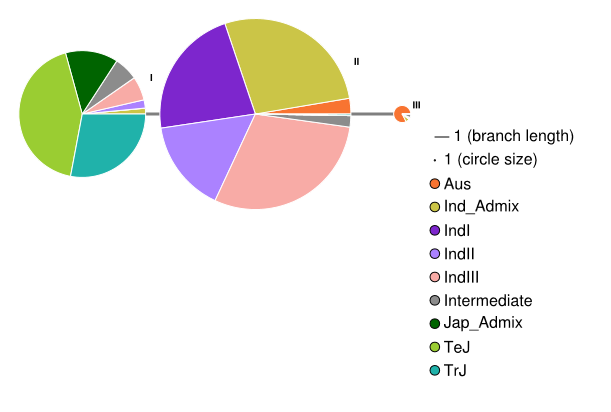

- **Os01t0799900 (*OsJaMC7)***

**
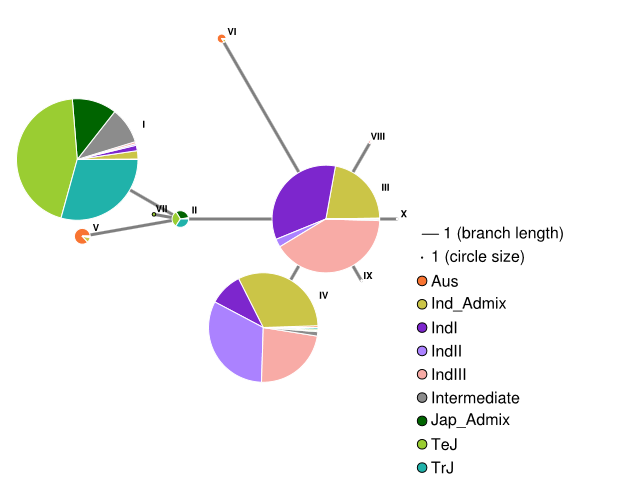
**

- **Os05t0496500 (*OsJaMC8*)**

**
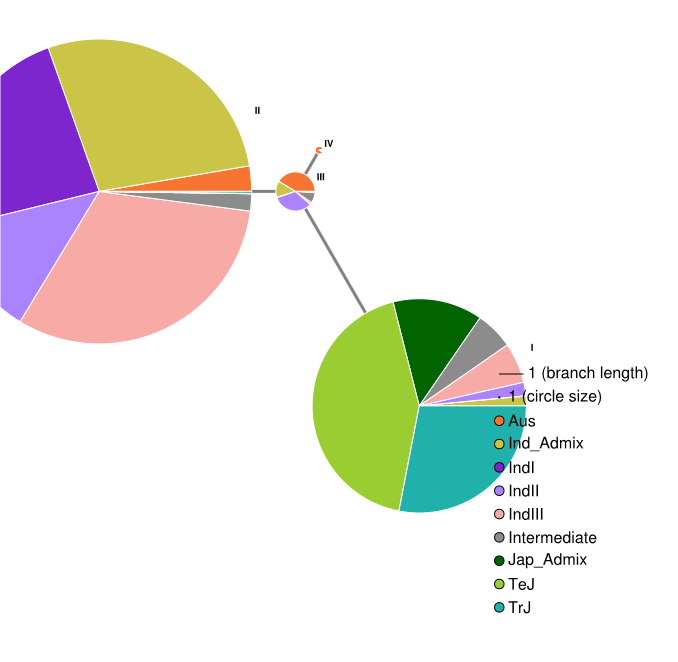
**

- **Os011t0134700 (*OsJaMC9)***

**
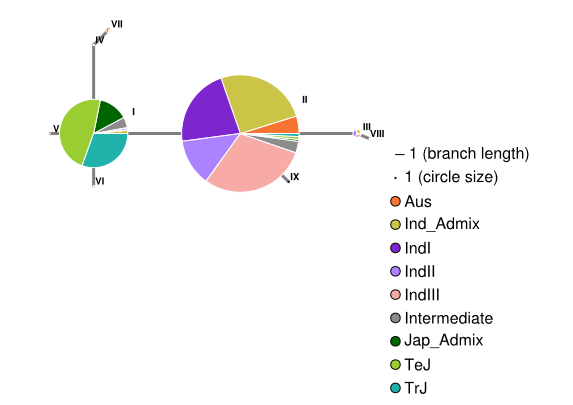
**

**Supplementary File S6.** Distribution and network of haplotypes identifed for the metacaspase genes in rice genome. The haplotypes were identified using whole genome resequencing data from 4,726 genotypes available at Rice Variation Map v2.0 (RiceVar Map 2.0).
