# Supplementary material for: Evolutionary Understanding of Metacaspase Genes in Cultivated and Wild Oryza Species and Its Role in Disease Resistance Mechanism in Rice
Source: Genes (Basel). 2020 Nov 26;11(12):1412. doi: 10.3390/genes11121412 (PMC7760854; doi:10.3390/genes11121412)
Supplement: Supplementary file 1 [file genes-11-01412-s001.zip › Supplementary Table 1.docx]

**Supplementary Table 1** Details of gene duplication types predicted for the 92 *matacaspase* genes identified in *Oryza* species

| ***Oryza* species** | **Type of Gene Duplication** | | | |
| --- | --- | --- | --- | --- |
|  | **Dispersal** | **Proximal** | **Tandem** | **Segmental Duplication** |
| ***O. barthii*** | 0 | 2 | 0 | 6 |
| ***O. brachyantha*** | 0 | 3 | 0 | 5 |
| ***O. glaberrima*** | 0 | 3 | 0 | 6 |
| ***O. glumaepatula*** | 0 | 2 | 0 | 6 |
| ***O. longistaminata*** | 0 | 4 | 0 | 4 |
| ***O. meridionalis*** | 7 | 0 | 0 | 0 |
| ***O. nivara*** | 0 | 3 | 0 | 5 |
| ***O. punctata*** | 0 | 3 | 0 | 5 |
| ***O. rufipogon*** | 0 | 3 | 0 | 6 |
| ***O. sativa indica*** | 0 | 2 | 0 | 6 |
| ***O. sativa japonica*** | 0 | 2 | 0 | 6 |
