# Supplementary figures and images for: Evolutionary Understanding of Metacaspase Genes in Cultivated and Wild Oryza Species and Its Role in Disease Resistance Mechanism in Rice
Source: Genes (Basel). 2020 Nov 26;11(12):1412. doi: 10.3390/genes11121412 (PMC7760854; doi:10.3390/genes11121412)

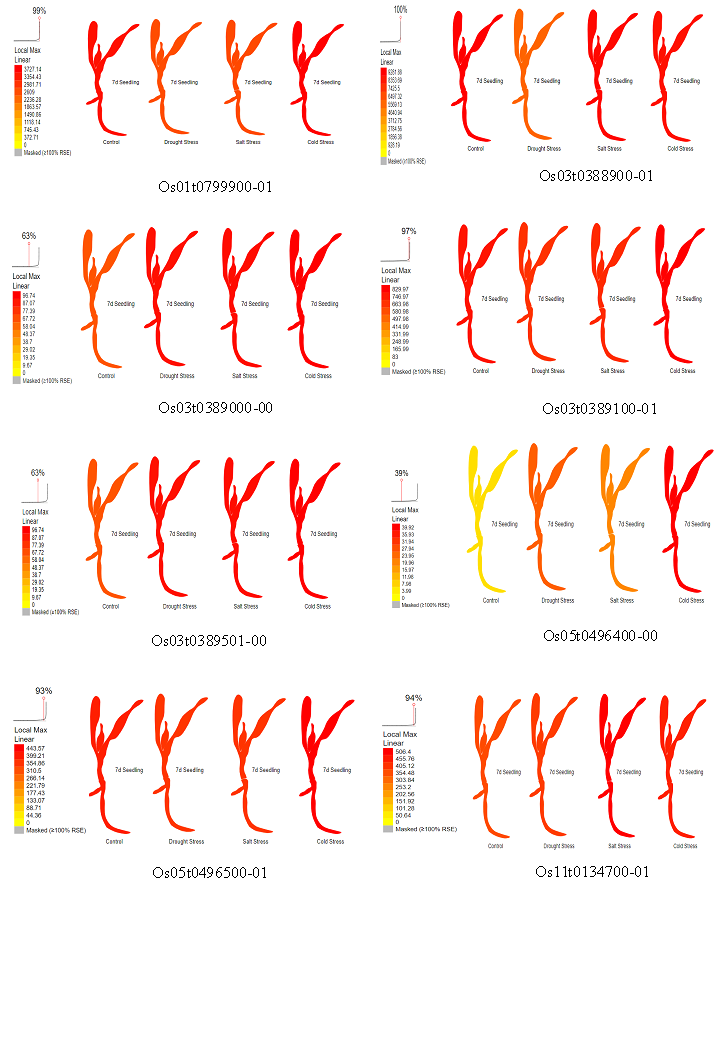


Fig: Representation of gene expression of *OsJaMCs* genes under different stress conditions

Supplement: Supplementary file 1 [file genes-11-01412-s001.zip › Supplementary File 8.docx]
